# Does time heal all wounds? Life course associations between child welfare involvement and mortality in prospective cohorts from Sweden and Britain

**DOI:** 10.1016/j.ssmph.2021.100772

**Published:** 2021-03-11

**Authors:** Josephine Jackisch, George B. Ploubidis, Dawid Gondek

**Affiliations:** a Centre for Health Equity Studies (CHESS), Karolinska Institutet/Stockholm University, Department of Public Health Sciences, Stockholm University, SE-106 91 Stockholm, Sweden; b International Max Planck Research School for Population, Health and Data Science, Max Planck Institute for Demographic Research, Konrad-Zuse-Str. 1, 18057 Rostock, Germany; c Centre for Longitudinal Studies, UCL Social Research Institute, University College London, 20 Bedford Way, London WC1H 0AL, United Kingdom; d Research Department of Epidemiology and Public Health, University College London1-19 Torrington Place, London, WC1E 7HB, United Kingdom

## Abstract

Child welfare involvement reflects childhood adversity and is associated with increased adult mortality, but it remains unclear how this association changes over the life course. Drawing on the Stockholm Birth Cohort Multigenerational Study (Sweden) and the National Childhood Development Study (Great Britain) this study examines whether inequalities within these cohorts diverge or converge. Involvement with child welfare services (ICWS) is divided into two levels (‘child welfare contact’ and ‘out-of-home care’). For each cohort, we quantify absolute health inequalities as differences in cumulative probabilities of death (18–58 years) and temporary life expectancy; and relative inequalities as hazard ratios in ten-year intervals and ratios of lifetime lost. Persistently, ICWS was associated with premature mortality. The strength of the association varied by age, sex and level of ICWS. Consistently across both countries, the most robust relationship was between out-of-home care and mortality, with statistically significant age-specific hazard ratios ranging between 1.8 and 3.4 for males and 1.8–2.1 for females. Child welfare contact that did not result in out-of-home placement showed less consistent results. Among females the mortality gap developed later compared to males. Estimates attenuate after controlling for family socioeconomic and other background variables but patterns remain intact. Our results show that absolute inequalities widen with increasing age, while relative inequalities might peak in early adulthood and then stabilize in midlife. The relative disadvantage among looked-after children in early adulthood is heightened by overall low rates of mortality at this age. Absolute inequality increases with age, highlighting the weight of the accumulation of disadvantage in mortality over time. The bulk of excess deaths that could be attributed to ICWS occurs from midlife onwards. Mechanisms that uphold the disadvantage after childhood experiences require further exploration. This study highlights that the association between out-of-home care and premature mortality seems to transcend welfare systems.

## Highlights

• Out-of-home care is associated with adult mortality across different welfare systems.
• We report age-dependent absolute and relative measures of inequality in mortality.
• Relative mortality risks seem to peak in early adulthood and to stabilize after.
• Absolute inequality continues to accumulate over the life course.
• The mortality gap emerges early in males and later in midlife among females.

## Introduction

Childhood adversity is “a leading cause of health inequality” according to the World Health Organization (Sethi et al., 2013, p. ix) and is associated with high societal costs that are considered largely avoidable (Bellis et al., 2019; Caspi et al., 2017; World Health Organization Regional Office for Europe, 2015). A growing body of literature has documented that children who experience different types of adversity have worse health (Bellis et al., 2019; Gilbert et al., 2009; Kalmakis & Chandler, 2015) and die earlier (Brown et al., 2009; Kelly-Irving et al., 2013; Rod et al., 2020). Among the large group of children who experience childhood adversity, those coming to the attention of child welfare services seem to have particularly accelerated mortality risks (Murray et al., 2020; Rod et al., 2020).

As most prospective studies of outcomes after child welfare involvement have a relatively short follow-up time, it remains unclear at what age inequality in mortality related to child welfare involvement emerges and whether this inequality increases or decreases over the life course. It could be expected that the influence of childhood adversity on health outcomes might wear off with time – as in the idiom ‘time heals all wounds’. Childhood inequalities might be outweighed by equalizing effects of policies and experiences in adulthood. In contrast, the cumulative inequality theory predicts that the association between childhood adversity and mortality will grow stronger with age (Ferraro et al., 2016). From a health inequalities perspective, we know that both conclusions might coexist depending on the choice of scale for measuring inequalities, as absolute or relative differences in mortality might return different results (Mehta et al., 2019).

Leveraging two maturing birth cohorts from Sweden and Great Britain (GB^1^), the principal aim of this study is to describe life course patterns of inequality in mortality associated with involvement with child welfare services (in the following, ICWS). Inequalities in mortality will be examined as both absolute differences in the probability of death and life expectancy, and relative ratios of death hazards and ratios of life years lost across the life course, between those with and without ICWS. Using data from Sweden and GB will help to elucidate whether ICWS is associated with premature mortality to a similar degree across different welfare systems.

### Absolute and relative inequalities in mortality from a life-course perspective

Social inequalities in mortality typically refer to differences in death between people in different socioeconomic positions, which are acquired and measured in adulthood (Mackenbach & Kunst, 1997). Such inequalities, however, may have childhood antecedents (Halfon et al., 2017; Hayward & Gorman, 2004). In this study, by inequality in mortality we refer to differences in adult premature mortality (age 18–58) between individuals with and without experiences of ICWS.

A general concern in research on health inequalities is whether differences in mortality between groups remain stable, converge, or diverge over time (Mackenbach et al., 2016; Rehnberg et al., 2019). The answer depends on the measurement scale chosen to assess inequalities (e.g. absolute or relative), and whether differences are assessed across historical time (over periods) or over age (over the life course). Over the life course, the relationship between many major risk factors and mortality is characterised by absolute increase and relative decrease over the life course (Mehta et al., 2019). The complementary information provided by absolute and relative measures of inequality has perhaps not yet received adequate attention in life course studies of premature mortality. While the calculation of (relative) hazard ratios (HRs) is widespread, measures of absolute differences are rarely used, yet they may be equally or more relevant to public health (Conner et al., 2019; Hernán, 2010).

There are theoretical grounds to believe that hazards are not stable over the life course. Based on the cumulative inequality theory (Dannefer, 2003; Ferraro et al., 2006), it can be assumed that initial differences in experiences of childhood adversity indicated by ICWS might lead to a divergence in mortality risks between groups with age (Dannefer, 2003). Other hypotheses, such as frailty theory, selective mortality, or the “age-*as*-leveller” hypothesis, predict convergence – a weakening of the relationship between early life risks and mortality over age (Hoffmann, 2011; Rehnberg et al., 2019). Both of these theories might be supported with increasing absolute and decreasing relative risks of mortality.

### Capturing childhood adversity through involvement with child welfare services

Despite being key to a better understanding of the associations between childhood adversity and health outcomes, prospectively measured indicators of childhood adversity and longitudinal follow-up of outcomes are rare (Gilbert et al., 2009; Spatz Widom et al., 2004). Nonetheless, many countries have child welfare or child protection systems in place that register reports, investigate possible neglect or maltreatment of children and provide services for families, which might not be able to care for their children. Recent studies show that ICWS and, in particular, being taken out of the family is associated with particularly accelerated risks of premature death in adulthood (Jackisch et al., 2019; Rod et al., 2020). Therefore, the study at hand uses ICWS as a proxy for childhood adversity. ICWS in this study is limited to contacts due to family-related circumstances excluding referrals only on the grounds of own behaviour (e.g. delinquency) of the child. ICWS was available in the Swedish and British cohorts from official records or parent-reported in childhood. For some families, ICWS means no measures or less intrusive measures are taken (e.g. warnings or regulations). For other families, ICWS results in the child being removed and placed in out-of-home care either in institutional or foster care. Consequently, it might be informative to distinguish between levels of involvement. ICWS has previously been found to be a strong predictor of mortality (Jackisch et al., 2019; Murray et al., 2020), but it remains unclear whether this can be generalized across countries and child welfare systems.

### Exploring two different contexts: Sweden and GB

In order to determine how sensitive ICWS is to differences in child welfare systems, we explore two countries: Sweden and GB. Each has been classified as a prototype of a child welfare system (Gilbert, 1997). Sweden, like all Nordic countries, has a family service orientation with the main intention to help families to alleviate problems that lead to household dysfunction. The long-term goal is uniting children with their families. In the 1950s–1970s, when the cohorts were young, Sweden considered out-of-home care an effective prevention tool, resulting in many short-term voluntary arrangements between parents and the state. Consequently, there is a high prevalence of out-of-home care in this cohort. Child welfare services in GB, as in most Anglo-Saxon countries, were oriented towards child protection with the primary aim of protecting the child against parental misbehaviour. Out-of-home care within this system was often implemented through coercive powers of the state and was likely to lead to long-term placements.

### Aim of the study

We aim to describe life course patterns of inequality in premature mortality associated with ICWS, using two longitudinal cohort studies: the Swedish 1953 Stockholm Birth Cohort Multigenerational Study (SBC Multigen) and the British 1958 National Child Development Study (NCDS). Inequalities in mortality are assessed in both absolute and relative terms between age 18 and 58. Childhood adversity is operationalized into two levels of ICWS. Since the prevalence of premature mortality differs across the sexes, males and females will be examined separately.

## Methods

### Sample and study design

**Fig. 1:**
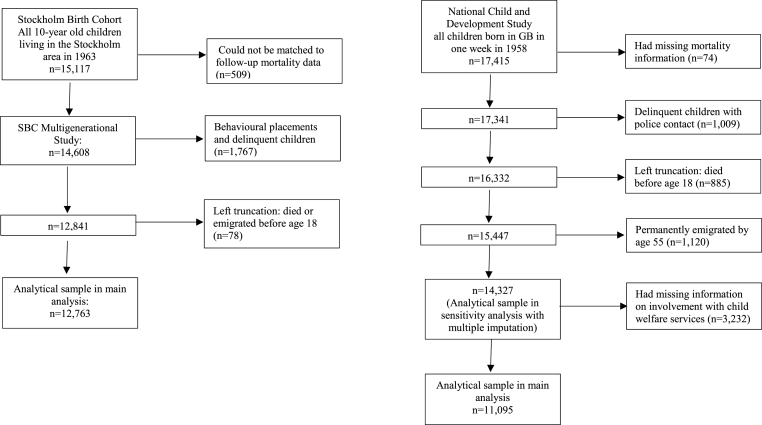
Samples.

The current study is based on data from two birth cohorts. The SBC Multigen includes individuals born in 1953, living in the greater Stockholm municipal area at the age of 10 and matched to follow-up register data (n = 14,608) (Almquist et al., 2019). The NCDS includes all live births during one week in 1958 (n = 17,415) in GB (Power & Elliott, 2006). Fig. 1 shows a more detailed overview of the selection of samples used in this study. The final analytic samples comprised 12,763 individuals from Sweden and 11,095 from GB.

### Variables

#### Involvement with child welfare services

ICWS is used to indicate childhood adversity. We separate two levels of severity of such involvement: contact with child welfare services that did not lead to taking the child out of the family (‘child welfare contact’) and substantiated investigations that led to the child being removed from their parents (‘out-of-home care’). Out-of-home care can be voluntary or involuntary placement in foster or institutional care. The reference group consists of children whose families have not been in contact with child welfare services (‘no child welfare’). Although child welfare services also respond to children's behavioural problems, this study focuses only on ICWS due to family-related circumstances. Children involved with child welfare due to own delinquency (or had problems with police), and had not previously been in contact with services due to family-related circumstances, were excluded (Sweden: n = 1767; GB: n = 1009) since those children have been shown to have accelerated mortality risks (Gao et al., 2017; Jackisch et al., 2019). Reports of ICWS were collected from local child welfare boards in Sweden and through interviews with parents throughout childhood in GB. Due to data availability, ICWS is captured from birth up to age 18 in the Swedish data and from birth up to age 16 in the British data (see Table 1 for details regarding the operationalization of variables and data sources in the two cohorts). While our information in Britain is last collected at age 16, it can be assumed that many of the family-related placements at age 16 where permanent and continue after that age. Therefore, and for consistency between cohorts, we start the mortality follow-up from age 18.

**Table 1:** Variable definitions.

	Sweden/The SBC Multigen		GB/The NCDS	
Variable	Age (year)	Definition and data source	Age (year)	Definition and data source
Mortality				
Premature all-cause mortality	18-58 (1971–2011)	Death from all causes, based on records in The Causes of Death Register	18-58 (1976–2016)	Death from all causes, based on death certificates and additional sources (Heywood, Johnson & Brown, 2015; Johnson & Brown, 2015)
Childhood adversity				
Involvement with child welfare services	0-18 (1953–1972)	Records on child welfare derived from local social registers in the Stockholm region, collected for three periods (ages 0–6, 7–12, 13–18)	0-16 (1958–1974)	Parental questionnaire (interviewed by health visitor) (ages 7, 11, and 16)
		‘No child welfare’: No record of being in contact with child welfare services		‘No child welfare’: No mention of having been in contact with child welfare services
		‘Child welfare contact’: Registration/investigation by child welfare services but no placement in out-of-home care		‘Child welfare contact’: Ever having required services from the Children's Department/Social Work Department, Dr. Barnardo's or other Children's Society, but not placed in out-of-home care
		‘Out-of-home care’: Ever placed in out-of-home care		‘Out-of-home care’: Ever been placed in out-of-home care
Sex	0 (1953)	Sex (male/female) of the cohort member at birth	0 (1958)	Sex (male/female) of the cohort member at birth

#### Mortality

The outcome variable is premature mortality from all causes between ages 18–58, i.e. covering the period 1971–2011 in the SBC Multigen and 1976–2016 in the NCDS. The information was derived from cause of death registers. In the NCDS, death data from death certificates and death cards was supplemented with information from follow-up cohort maintenance work (Johnson & Brown, 2015). We quantify inequalities in mortality as differences between children with and without ICWS regarding age-specific HRs of death, temporary life expectancy, and life years lost (between ages 18–58).

#### Sex

Previous research has not reported consistent findings regarding sex differences in the prevalence of childhood adversity (Petruccelli et al., 2019). It is nevertheless well known that males have increased risks of premature mortality compared to females. We use the biological sex as recorded at birth of the cohort member (male or female) as a stratifying variable throughout the main analyses (both sexes are pooled in the sensitivity analysis).

### Analytic approach

Analyses were conducted separately for the two cohorts from Sweden and GB. The outcome was defined as time-to-event (death), presenting unadjusted results in the main analysis and adjusted results in subsequent sensitivity analysis.

#### Absolute differences in mortality

Life tables were calculated and stratified by level of ICWS. The results were plotted as cumulative failure graphs, which illustrate the probability of death by age for the different levels of ICWS – note that the measure is cumulative over age. Absolute risks of mortality capture both the baseline hazard of dying by age among those without ICWS (see failure curve of the ‘no child welfare’ group) and the excess death risks in the child welfare populations (‘child welfare contact’ and ‘out-of-home care’). Risk tables that show numbers of population at risk at age 18, 28, 38, 48 and 58 accompany each graph.

To aid visualisation of results, a separate panel quantifies the cumulative absolute inequalities in life expectancy between ages 18–58. This was calculated as differences in temporary life expectancy, defined as the average number of person-years lived in the given interval per person from age 18; differences between groups are calculated as ‘child welfare contact’ or ’out-of-home care’ minus ‘no child welfare’. Negative values, thus, indicate a loss in life expectancy among groups with experiences of ICWS.

#### Relative differences in mortality

Relative measures of risk express the risk of a disadvantaged group as a ratio of the risk of an advantaged group. We compare groups with and without ICWS, calculating age interval-specific HRs of death in a series of discrete time piecewise constant models, e.g., a HR of two means that the group had double the risk of death than the reference group on average in the specified ten-year age interval. Age was split into the intervals 18–27, 28–37, 38–47, and 48–58 and crude HRs were plotted for each level of ICWS.

As an additional relative measure, we calculated the ratio of the life years lost cumulatively over an increasingly long age interval, which represents the area under the failure function from age 18 up to ages 28, 38, 48, and 58. Differences in life years lost were computed here as the ratio between ‘child welfare contact’ or ‘out-of-home care’ divided by ‘no child welfare’, e.g., a ratio of two means that the group had double the life expectancy losses than the reference group up to the specified upper age.

#### Sensitivity analyses

Sensitivity analyses estimated the absolute and relative risk of death across groups controlling for various potential confounding factors (maternal age, marital status, birth weight, and socioeconomic status of the family) and indicators for household dysfunction as measured by parental substance use, mental health problems, criminality, divorce, and death (see supplementary Table 1). Household dysfunction might indicate adversities that precede ICWS and are likely to contribute to premature mortality. Laplace regression was used to analyse the absolute differences in survival time (expressed in years) between groups at the 5th survival percentile (this percentile was used to avoid extrapolation beyond the range of observed data) (Bellavia et al., 2015). Cox models were used to model relative risks of mortality. Additional robustness checks used multiple imputation by including participants with missing information on ICWS (n = 3232) in the British sample.

## Results

### Descriptive statistics

The distribution of mortality in the SBC Multigen and the NCDS is presented in Table 2 stratified by sex and the level of ICWS (e.g. ‘no child welfare’, ‘child welfare contact’, and ‘out-of-home care’). Approximately 5% of both cohorts died between ages 18–58. In both countries, 11% of the cohorts had experiences of ICWS, while incidence of out-of-home care during childhood was higher in Sweden (8%) than in GB (5%).

**Table 2:** Descriptive statistics stratified by sex and child welfare involvement.

Sweden/SBC Multigen						
	Males			Females		
	No child welfare (n = 5338; 88%)	Child welfare contact (n = 174; 3%)	Out-of-home care (n = 548; 9%)	No child welfare (n = 6050; 90%)	Child welfare contact (n = 162; 2%)	Out-of-home care (n = 491; 7%)
Number of deaths (age 18–58)	293	22	65	225	12	34
Proportion of deaths % (age 18–58)	5.49	12.64	11.86	3.72	7.41	6.92
**GB/NCDS**						
	**Males**			**Females**		
	No child welfare (n = 4845; 89%)	Child welfare contact (n = 336; 6%)	Out-of-home care (n = 253; 5%)	No child welfare (n = 5051; 89%)	Child welfare contact (n = 339; 6%)	Out-of-home care (n = 271; 5%)
Number of deaths (age 18–58)	304	34	32	231	16	24
Proportion of deaths % (age 18–58)	6.27	10.12	12.65	4.57	4.72	8.86

### The mortality gap over the life course

**Fig. 2:**
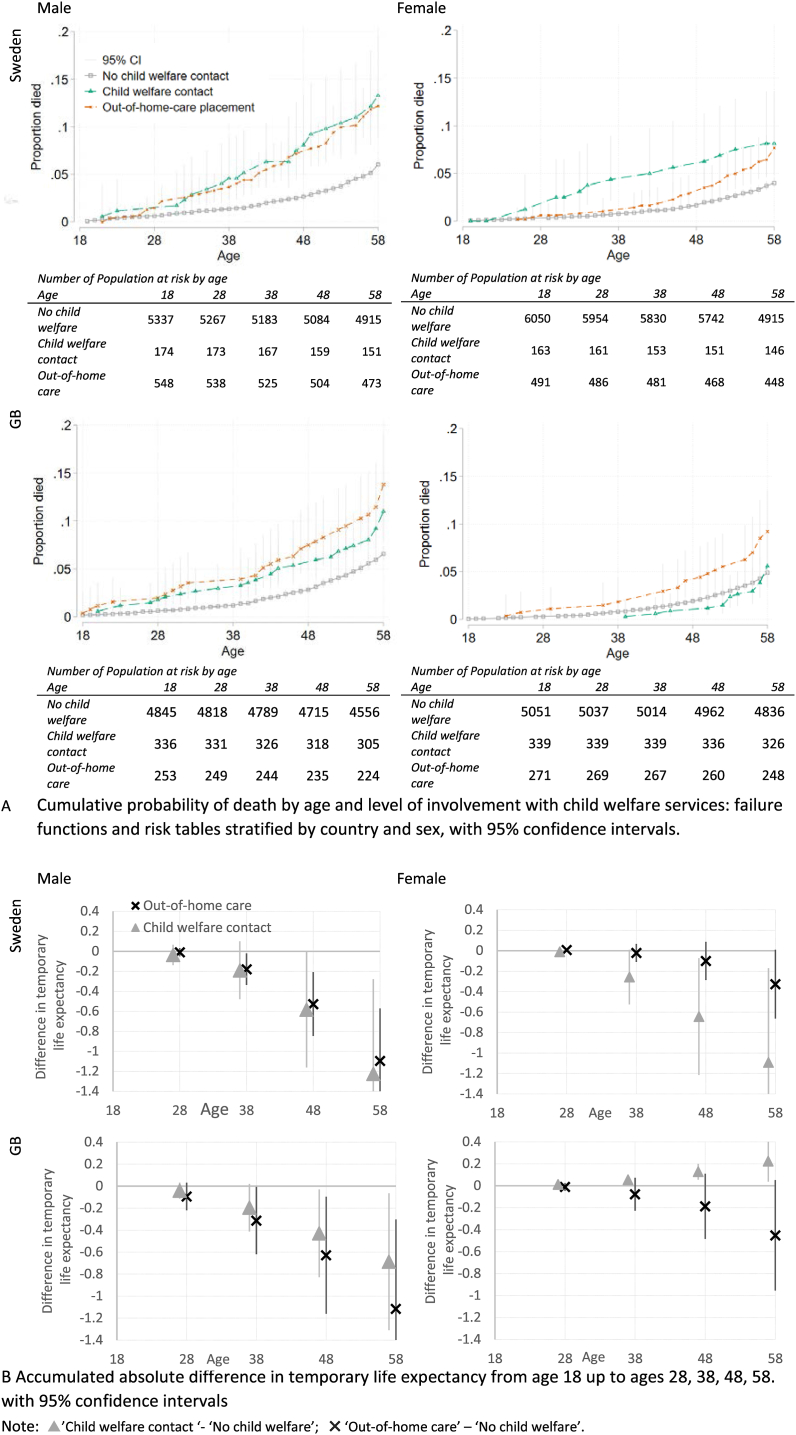
Absolute differences in mortality over the life course by country and sex.

Fig. 2 shows the absolute risks of mortality by level of ICWS by age: in Panel A in terms of probabilities of death and in Panel B in terms of absolute difference in temporary life expectancy between child welfare groups and the reference group. Those with ICWS have higher risk of death across the life course compared to those without such experiences. For instance, at age 35 had 1/1% (in Sweden/GB, respectively) of males without ICWS died, versus 4/3% of males with child welfare contact and 3/4% of males with out-of-home care. In comparison, at age 55 had 5% of those without ICWS died, versus 11/8% of those with child welfare contact and 11/10% of those with out-of-home care (Fig. 2, panel A). The absolute difference in life expectancy between ‘out-of-home care’ and ‘no child welfare’ accumulates over the life course, culminating in over one year of life lost on average by the age of 58 in males and almost five months lost in females (Fig. 2, panel B).

**Fig. 3:**
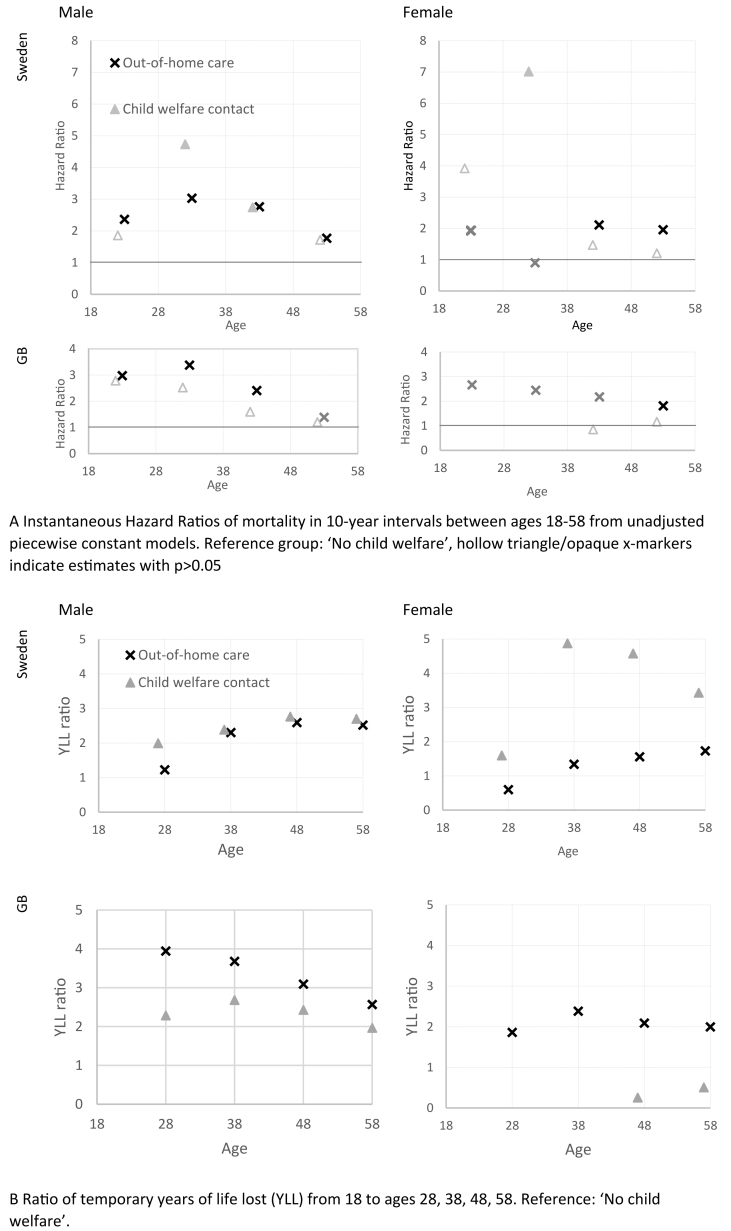
Relative inequality in mortality and survival over the life course by country and stratified by sex.

Fig. 3 illustrates the relative risk of death by age: Panel A reports the age group-specific HRs from discrete constant models, and Panel B shows the relative ratio of temporary life years lost. The most robust relationship was between ‘out-of-home care’ and mortality, with persistent age-specific HRs ranging between 1.4 and 3.4 for males, of which HRs between 1.8 and 3.4 were statistically significant. For females the according HRs range between 0.9 and 2.7 but suffer from high uncertainty; statistically significant HR range between 1.8 and 2.1 for females (Fig. 3, panel A). Due to low incidence of death, HRs for the age group 18–28 have large confidence intervals. Among males, results show the highest instantaneous HRs are for men with ICWS between age 28–37, with three-fold increased hazards for ‘out-of-home care’ and HRs ranging from 2.5 (GB) to 4.7 (Sweden) for ‘child welfare contact’. This means that those with out-of-home care experiences had triple the risk of dying between ages 28–37 than the reference group (Fig. 3, panel A). The peak is followed by declining piecewise HRs and a stabilizing (Sweden) or declining (GB) cumulative ratio of temporary life years lost (Fig. 3, panel B). For British females, the general trends are similar although with slightly lower effect sizes and higher uncertainty. Swedish females in ‘out-of-home care’ form an exception to this pattern as their relative mortality risks peak slightly later (age 38–47) (Fig. 3, panel A and Fig. 2, panel A). Overall, the relative risks of death for ‘out-of-home care’ do not converge to the level of ‘no child welfare’ within the observation window, although in GB estimates seem to suggest further decline in older ages (Fig. 3, panel A).

### Differences between countries

Out-of-home care is associated with increased mortality risks similarly in GB and in Sweden: by the age of 58 over 10% of males with ICWS and 7–9% of females with ‘out-of-home care’ had died, while it was only about 4–6% of the reference males and females (Fig. 2, panel A). There is no difference in survival between the two levels of ICWS among Swedish males, whereas British males have slightly higher risks of mortality if they had ‘out-of-home care’ compared to ‘child welfare contact’ (Fig. 2, panel A). HRs for ‘out-of-home care’ are slightly higher in early adulthood in GB than in Sweden (Fig. 3, panel A).

### Sex differences

There are notable sex differences in when the gap in mortality emerges. For males, a gap in mortality is already apparent in their 30s (Fig. 2, panel B and Fig. 3, panel B). Females in ‘out-of-home care’, however, have a similar pattern of mortality as ‘no child welfare’ up to age 40, after which a mortality gap seems to emerge (Fig. 2, panel B). For females, the less severe level of ‘child welfare contact’ showed different patterns than for males and different patterns across the two countries. In Sweden, females with ‘child welfare contact’ appear to have higher mortality risks than those in ‘out-of-home care’, whereas in GB, they seem to have more favourable outcomes with no difference from the reference group. All estimates, but in particular those of females with ‘child welfare contact’, suffer from large uncertainty as there are very few cases of premature mortality.

### Robustness of results

Sensitivity analyses estimated the absolute and relative mortality risks in two steps. First, we controlled for known family background factors that could confound the relationship between ICWS and mortality. Second, in a fully adjusted model, indicators of household dysfunction were added as we hypothesize that they would explain a substantive part of the adversity that is indicated by ICWS. Low birthweight (<2500 g), manual socioeconomic family background, teenage and unmarried mothers, and all household dysfunctions were more common in those with compared to those without ICWS (Supplementary Table 2). The magnitude of the mortality risks attenuated slightly after adjustment for background factors and more so in the fully adjusted models, but the overall associations and patterns remained robust (supplementary Table 3). Similar results were found in multiple imputed data from GB that included the n = 3232 individuals, who had missing information on the exposure.

## Discussion

We investigated the association between childhood adversity and premature mortality across the ages 18–58 in a Swedish and a British cohort. The results showed that childhood adversity – here indicated by ICWS – is associated with increased risks of mortality in a way that persists across adulthood. This is in line with the relatively few previous studies on mortality after ICWS, which have reported higher all-cause mortality among child welfare recipients when leaving care (Kalland et al., 2001; Thompson & Newman, 1995), in early adulthood (<age 27) (Hjern et al., 2004; Vinnerljung & Ribe, 2001) and into midlife (Murray et al., 2020). A study using the same Swedish data set as used in this study, has documented that the mortality disadvantage extends up to entry into retirement age (Jackisch et al., 2019). We expand this work by replicating these findings with data from GB. The knowledge derived from this study might open new paths for understanding the origins of premature mortality.

### Mortality risks over the life course: accumulating and also stabilizing after an initial peak

Absolute mortality risks associated with ‘out-of-home care’ were substantive and continued to increase as cohorts got older. This means that the bulk of excess deaths among those with experiences of child welfare occurred in midlife. The results on absolute inequality can be interpreted from the perspective of cumulative inequality theory. Rooted in the concept of cumulative advantage/disadvantage, a basic tenet for this theory is that “disadvantage accumulates over the life course, thereby differentiating a cohort over time” (Dannefer, 2003; Ferraro & Shippee, 2009, p. 334). The exponential increase of absolute differences through adult life would, according to this theory, be explained by early adversities increasing the exposure to other risks, limiting agency and available resources, and thereby contributing to an accumulation of inequality (Ferraro, 2011). As anticipated, the answer to the question of age-dependency differed on whether mortality risks were assessed as absolute differences or as relative ratios.

In terms of relative risks, the results show a relative stability of risks. In comparison to the risk of mortality in those without experiences of ICWS, populations with ICWS were the most disadvantaged in early adulthood (ages 28–38). From a health inequality perspective, this is not very surprising as it has been shown that relative inequalities tend to be bigger at low levels of mortality (Houweling et al., 2007), and in early adulthood levels of mortality tend to be low compared to other age groups (particularly among females). Despite a decline in immediate risk ratios in midlife, the results indicate a relative stability of risks up to the age of 58 (at least for ‘out-of-home care’). The age-trends in British males might indicate the possibility of a convergence of risks. The possibility of a convergence of risks is inconclusive here and might be due to other reasons. This should be further explored with longer follow-up times, as studies that have documented convergence for other risk factors usually find it at later ages (Hoffmann, 2011; Mehta et al., 2019; Rehnberg et al., 2019). In accordance with earlier studies (Mehta et al., 2019), we thus find that absolute and relative inequalities show complementary pictures.

### The mortality gap emerges at different age for male and females

Our results also show that the inequality gap emerges later for females than for males. The gender gap in mortality is a well-researched topic and males tend to have higher mortality due to violence, accidents and behaviour-related causes particularly in early adulthood (Sundberg et al., 2018; Trovato & Heyen, 2006). Such causes are similar to causes that have been documented in relation to childhood adversity namely suicide, external causes and other avoidable causes (Almquist et al., 2020; Hjern et al., 2004; Murray et al., 2020; Putnam-Hornstein, 2011). Causes of death among females might be more related to chronic conditions and mental health related causes which are more common in females and tend to occur later (Whiteford et al., 2013).

### Involvement with child welfare services: a reliable indicator of adversity?

In the quest for reliable prospective measures of childhood adversity, we suggested ICWS to be a useful indicator. A major strength is that it is often routinely recorded during childhood. Even when reported by parents, like in the British sample, such information might be more factual and thus less prone to affective bias compared to questions about childhood experiences of e.g. abuse, neglect, or parental mental illness. We theoretically derived two levels of ICWS: ‘child welfare contact’ and ‘out-of-home care’, which allowed us to reflect in a more nuanced way on the usefulness of ICWS in the two countries. Although the aim of measures taken by child welfare services is to improve children's life chances, studies have consistently shown that educational outcomes, employment, and health are generally worse for children with out-of-home care experiences (Kääriälä & Hiilamo, 2017), even compared to siblings who remained with the biological parents (Brännström et al., 2020). Therefore, ‘out-of-home care’ could be expected to indicate the most severe level of adversity. At the same time, removing a child from home is meant to improve life chances and it is difficult to know whether the exposure might be even more severe for those staying in the family. Our results show that both levels of ICWS are associated with premature mortality (with exception of British females in contact with child welfare).

### Comparing results across countries

The results for ‘out-of-home care’ were strikingly similar across Sweden and GB. On the one hand, this is surprising given the differences in welfare systems, including differences in selection into child welfare. Living through the golden years of the Swedish welfare state throughout adulthood, the Swedish welfare system would be expected to modify the association at least partly. On the other hand, the underlying exposure that led services to intervene would be expected to be equally damaging to children's future health irrespective of country context. Several studies have shown that severe stress in childhood can have implications for biological and psychological development (Barboza Solís et al., 2015; Castagné et al., 2018; Ploubidis et al., 2020), which in turn could be associated with premature mortality. Our findings support that out-of-home care is reliably associated with disadvantages in survival in both countries.

The group with child welfare contact was more sensitive to country context. This level of exposure is less well defined in comparison to ‘out-of-home care’: the underlying adversities reflected in this group are likely to be different across countries, which might explain why the incidence and results also differ. The child welfare authority administers all services in Sweden, where the majority of cases of child welfare contact come from mandatory reporting of suspected adverse circumstances in the family. While in GB there is a greater involvement of child welfare charities, which may differ in their objectives and scope of work. Hence, ‘child welfare contact’ in GB might include a more diverse group compared to Sweden, for instance in terms of severity or source of problems.

### Strengths and limitations

The main strength of our study is that we use prospective birth cohorts with long-term mortality follow-ups. Prospectively measured variables have the advantage of reflecting the temporal order of events. The majority of previous studies on childhood adversity have relied on samples recruited in adulthood and retrospectively reported information. Such designs might lead to false conclusions, for instance due to survival bias, measurement bias due to recall, and the inability to control for confounders (Jivraj et al., 2020). While the Swedish data was conditional on survival until age 10, attrition in the follow-up is very low due to the national registers. The British data had some more attrition and non-response, which limits this study. Disadvantaged families are less likely to participate in studies and more likely to be lost to follow-up. We were, however, able to find consistent results in our sensitivity analysis on the British data by multiple imputation including those that had missing data on the exposure (n = 3233) (Supplementary Table 3).

The birth cohorts used in our study are deemed to be representative for individuals born in respective countries in the 1950s and 1960s. However, further research is needed to infer whether the association between ICWS and mortality remains in the younger birth cohorts – in the face of changing welfare systems and societal contexts. Moreover, despite efforts to harmonize the analytic approach across the two cohorts, there are differences in the data between Sweden and GB: we used administrative records (up until age 18) from the SBC Multigen and self-reported child welfare data (up until age 16) from the NCDS. It is, however, likely that placements did continue after the date of interview in Britain and most newly initiated placements in Sweden between ages 16–18 were due to behavioural reasons (e.g. delinquency).

A weakness of the ICWS measure is that it does not allow for distinguishing actual experiences of adversity with the social workers’ judgement of severity of these experiences (Cocozza & Hort, 2011). The data was neither detailed enough to investigate whether the associations were sensitive to the age of the child at ICWS, nor to account for chronicity of the experience. The question of timing and sensitive periods for ICWS is relevant from a life course perspective and could potentially be tested with more detailed data from younger cohorts. As much secondary data, child welfare registrations lack specificity. Arguably, children involved with child welfare services will have experienced some type of childhood adversity, but registers seldom provide reliable information on type, duration, or frequency of adversity. Since this study is based in observational data, bias due to unmeasured confounding cannot be ruled out and it is not possible to draw causal inferences on the potential effects of ICWS.

While we cannot infer a causal effect of ICWS on mortality with certainty, the results show that in both investigated child welfare systems, the state intervention has not been successful in equalizing chances for children. This raises questions about the potential mechanisms in adulthood that contribute to accumulating risks among those with experiences of childhood adversity. Knowing more about the causes of the excess deaths might provide more clarity about potential entry points for prevention of premature mortality in this vulnerable population. Neither of these questions could be answered within the scope of this study. Moreover, it is conceivable that there are differences in the general population pattern of all-cause mortality between the two countries and potential country-specific period effects on mortality (e.g. due to effects of the HIV epidemic, economic recessions, or changes in legislation encountered at different times) which we were unable to detect in this study due to limited sample size and using single birth cohorts.

Future studies should explore mechanisms that might uphold the disadvantage. More research is also needed to validate the viability of routinely collected data for life course studies. Comparative studies across countries and studies evaluating policies through natural experiments are still rare in this field but might be important to answer the question regarding whether child welfare services could possibly mitigate the life chances of vulnerable populations.

## Conclusions

Prior research has documented the health consequences of childhood adversity. This study is, to the best of our knowledge, the first to describe absolute and relative mortality risks among people involved with child welfare services and how this relationship varies over the life course. Our analyses show that health inequalities associated with out-of-home care are substantial and persistent. Absolute and relative mortality risks convey complementary messages of relative susceptibility among child welfare populations to early adult deaths and a significant absolute burden of premature mortality from midlife onwards. High-risk groups for premature mortality can already be identified in childhood both in Sweden and in Great Britain.

## Author contributions

Contributors: JJ and DG planned and designed the study and analyzed the data. GP advised on methodology. JJ drafted the original manuscript, JJ and DG reviewed and edited the final draft. JJ, DG and GP interpreted the results and reviewed and revised the manuscript and approved the final manuscript for submission. JJ attests that all listed authors meet authorship criteria and that no others meeting the criteria have been omitted.

## Declaration of competing interest

None.

## Footnotes

1 **Abbreviations used in this article:** GB, Great Britain; HR, hazard ratio; ICWS, involvement with child welfare services; NCDS, National Childhood Development Study; SBC Multigen, Stockholm Birth Cohort Multigenerational Study; YLL, partial life years lost.
