# Supplementary material for: Does time heal all wounds? Life course associations between child welfare involvement and mortality in prospective cohorts from Sweden and Britain
Source: SSM Popul Health. 2021 Mar 11;14:100772. doi: 10.1016/j.ssmph.2021.100772 (PMC8010861; doi:10.1016/j.ssmph.2021.100772)
Supplement: Supplementary file 1 — Multimedia component 1 [file mmc1.docx]

Supplementary Table 1. Additional control variables used in sensitivity analysis.

|  | Sweden/The SBC Multigen | | GB/NCDS | |
| --- | --- | --- | --- | --- |
| Variable | Age (year) | Definition and data source | Age (year) | Definition and data source |
| Other variables measured at birth of the cohort member | | | | |
| Birth weight | 0 (1953) | As recorded in delivery journals. | 0 (1958) | recorded in the birth survey, which was completed by a midwife who attended the delivery |
| Socioeconomic family background | 0 (1953) | Father’s* occupational class, based on Delivery records, or retrieved from the 1953 population register and the parish books of births. Dichotomized into manual/non-manual | 0 (1958) | Father’s occupational class, coded according to the classification Socio-economic Groups (SEG) classification introduced in 1951. Dichotomized into manual/non-manual |
| Mother’s age | 0 (1953) | As recorded in delivery journals and population register | 0 (1958) | Self-reported by mother at birth |
| Marital status of mother | 0 (1953) | As recorded in delivery journals and Census 1960 | 0 (1958) | Self-reported by mother at birth |
| Household dysfunction | | | | |
| Incarceration | 0-18 (1953-1972) | Father having a record of incarceration or probation with the National Police Board | 7, 11, 16 (1965, 1969, 1974) | Family having required the services of Probation Officer or Police; reported by parents |
| Divorce | 0-7 (1953-1960) | Parental divorce, based on the Census 1960 and The Population Register from 1953 | 7 (1965) | Parental divorce, separation or desertion; reported by parents |
| Death | 0-7 (1953-1960) | Parental death, based on The Cause of Death Register and The Multigeneration Register | 7 (1965) | Death of child's father or mother; reported by parents |
| Alcohol problems | 0-7 (1953-1960) | Parental alcohol problems registered by local temperance committees, The Social Register or at least two drunk driving related sentences, based on National Police Board Data | 7 (1965) | Parental alcohol problems; reported by parents |
| Mental health problems | 0-12 (1953-1965) | Parental mental health problems or psychiatric disease, noted in The Social Register | 7, 11 (1965, 1969) | Parental mental health problems; reported by parents |

*Or step/adoptive father’s. In some cases, mother’s occupational status was used instead, namely, if the mother was single and not cohabiting in 1953 and was still single in 1957, if the father's or stepfather's occupation was missing, or if the mother had an upper-class or upper-middle-class occupation whereas the father was a worker.

Supplementary Table 2. Descriptive statistics of potential confounders

| Sweden/The SBC Multigen | Stratified by involvement with child welfare services | | |
| --- | --- | --- | --- |
|  | No child welfare  (n=11,388; 89%) | Child welfare contact  (n=336; 3%) | Out-of-home care  (n=1,039; 8%) |
| Household dysfunction: | % | % | % |
| Alcohol | 0.90 | 12.80 | 7.89 |
| Divorce | 2.67 | 10.71 | 13.38 |
| Mental illness | 1.83 | 25.00 | 24.93 |
| Crime | 3.55 | 18.45 | 16.75 |
| Death | 0.30 | 1.49 | 2.41 |
| Manual background | 44.13 | 66.07 | 65.54 |
| Mother’s age <20 years | 3.63 | 5.06 | 9.62 |
| Unmarried mother | 4.14 | 15.77 | 23.97 |
| Low birth weight (<2500gr) | 2.13 | 4.17 | 6.06 |
| **GB/NCDS** | **Stratified by involvement with child welfare services** | | |
|  | No child welfare  (n=9,896; 89%) | Child welfare contact  (n=675; 6%) | Out-of-home care  (n=524; 5%) |
| Household dysfunction: | % | % | % |
| Alcohol | 0.67 | 2.91 | 5.28 |
| Divorce | 6.22 | 16.06 | 21.30 |
| Mental illness | 3.20 | 16.15 | 21.18 |
| Criminality | 3.74 | 20.00 | 18.89 |
| Death | 1.37 | 1.84 | 4.90 |
| Manual background | 71.86 | 84.49 | 90.65 |
| Mother’s age <20 years | 4.43 | 10.22 | 11.64 |
| Unmarried mother | 2.24 | 10.81 | 21.18 |
| Low birth weight (<2500gr) | 5.13 | 7.26 | 10.88 |

Supplementary Table 3. Sensitivity analysis of relative and absolute risks of premature death: gender adjusted and fully adjusted models.

|  | Sweden: SBC Multigen (n = 12,450) | | | | | | | |
| --- | --- | --- | --- | --- | --- | --- | --- | --- |
|  | Sex adjusted | |  | Adjusted for background^a^ | |  | Fully adjusted^b^ | |
| **Relative: Cox Model** | HR | 95% CI |  | HR | 95% CI |  | HR | 95% CI |
| Child welfare contact | 2.24 | 1.57-3.18 |  | 2.10 | 1.47-3.00 |  | 1.86 | 1.28-2.71 |
| Out-of-home-care | 2.13 | 1.71-2.64 |  | 1.94 | 1.54-2.44 |  | 1.79 | 1.40-2.30 |
| **Absolute: Laplace quintile regression** | B Q.05* | 95% CI |  | B Q.05* | 95% CI |  | B Q.05* | 95% CI |
| Child welfare contact | -17.89 | -26.29;-9.49 |  | -15.37 | -22.89;-7.86 |  | -14.95 | -21.60;-8.30 |
| Out-of-home-care | -12.07 | -15.86;-8.28 |  | -10.53 | -14.63;-6.43 |  | -7.85 | -11.87;-3.84 |
|  | **Great Britain: NCDS (n = 9,463)** | | | | | | | |
|  | Sex adjusted | |  | Adjusted for background^a^ | |  | Fully adjusted^b^ | |
| **Relative: Cox Model** | HR | 95% CI |  | HR | 95% CI |  | HR | 95% CI |
| Child welfare contact | 1.13 | 0.77-1.66 |  | 1.11 | 0.75-1.63 |  | 1.04 | 0.70-1.53 |
| Out-of-home-care | 1.97 | 1.41-2.77 |  | 1.89 | 1.33-2.69 |  | 1.64 | 1.14-2.37 |
| **Absolute: Laplace quintile regression** | B Q .05* | 95% CI |  | B Q.05* | 95% CI |  | B Q.05* | 95% CI |
| Child welfare contact | -0.25 | -5.59;5.08 |  | 0.03 | -5.22;5.29 |  | 0.09 | -5.64;5.82 |
| Out-of-home-care | -7.22 | -9.48;-4.95 |  | -6.08 | -8.72;-2.78 |  | -4.54 | -8.27;-0.81 |
| Additional sensitivity analysis adding the individuals lost to follow-up | **Great Britain: NCDS (n = 14,327)** based on multiple imputation | | | | | | | |
|  | Sex adjusted | |  | Adjusted for background^a^ | |  | Fully adjusted^b^ | |
| **Relative: Cox Model** | HR | 95% CI |  | HR | 95% CI |  | HR | 95% CI |
| Child welfare contact | 1.36 | 1.03, 1.80 |  | 1.30 | 0.98, 1.73 |  | 1.24 | 0.91, 1.67 |
| Out-of-home-care | 1.76 | 1.35, 2.33 |  | 1.68 | 1.27, 2.22 |  | 1.58 | 1.17, 2.12 |
| **Absolute: Laplace quintile regression** | B Q .05* | 95% CI |  | B Q.05* | 95% CI |  | B Q.05* | 95% CI |
| Child welfare contact | -3.64 | -14.73, -2.77 |  | -2.95 | -7.96, 2.06 |  | -2.71 | -8.21, 2.79 |
| Out-of-home-care | -8.75 | -8.56, 1.27 |  | -7.71 | -13.24, -2.19 |  | -6.19 | -11.61, -0.77 |

* Laplace regression coefficient for the 5^th^ percentile of survival can be interpreted as the absolute difference in years (underlying scale is age) at which 5% of the respective group has died (controlled for other predictors)

^a^ adjusted for birth weight, socioeconomic family background, mother’s age and marital status of mother

^b^ adjusted for birth weight, socioeconomic family background, mother’s age and marital status of mother, as well as household dysfunction (parental incarceration, divorce, death, alcohol problems and mental health problems)
